# Expression analysis and clinical significance of eIF4E, VEGF-C, E-cadherin and MMP-2 in colorectal adenocarcinoma

**DOI:** 10.18632/oncotarget.13453

**Published:** 2016-11-19

**Authors:** Minna Gao, Xiong Zhang, Dan Li, Ping He, Wenguang Tian, Bo Zeng

**Affiliations:** ^1^ Department of Pathology, Chongqing Medical University, Chongqing 400016, China; ^2^ College of Basic Medicine, Chongqing Medical University, Chongqing 400016, China; ^3^ Department of Critical Care Medicine, Yongchuan Hospital, Chongqing Medical University, Chongqing 402160, China; ^4^ Department of Infectious diseases, Yongchuan Hospital, Chongqing Medical University, Chongqing 402160, China; ^5^ Department of Gastroenterology, Yongchuan Hospital, Chongqing Medical University, Chongqing 402160, China

**Keywords:** colorectal cancer, eIF4E, VEGF-C, MMP-2, E-cadherin

## Abstract

The underlying mechanisms of colorectal carcinoma (CRC) metastasis remain to be elucidated. The aim of this study is to investigate clinical significance and the expression of eIF4E, VEGF-C, MMP-2, and E-cadherin in the CRC metastasis. We investigated their expressions in 108 patients, analyzed the relationships between their expressions in CRC and evaluated the relationships between their expressions and clinical pathogenic parameters. Furthermore, their roles in patient survival and in CRC metastasis were also investigated. We found that eIF4E, VEGF-C and MMP-2 were up-regulated in CRC, and their expression frequencies (EFs) were higher in cancerous tissues than in adjacent normal tissues. The EF of E-cadherin is lower in cancerous tissues than in adjacent normal tissues. Totally, their EFs were not associated with sex and age of patient, however, their EFs were associated with tumor differentiation, the depth of invasion, lymph node metastasis and tumor stages. Furthermore, eIF4E, VEGF-C, and MMP-2 shortened and E-cadherin prolonged survival in patient-derived CRC xenografts. Similarly, eIF4E, VEGF-C, and MMP-2 promoted and E-cadherin suppressed the lung metastasis of CRC cells. In addition, knockdown of eIF4E inhibited migration of CRC cells, downregulated VEGF-C, MMP-2 and upregulated E-cadherin. In conclusion, eIF4E promoted CRC metastasis via up-regulating the expression of VEGF-C, MMP-2 and suppressing E-cadherin.

## INTRODUCTION

Colorectal carcinoma is the third most common malignant tumor in both men and women [1]. The metastasis and recurrence are the leading cause for death of patients with the colon cancer. The identification of novel biomarkers or therapeutic target during colorectal cancer progression, especially during the metastasis and recurrence, can effectively devote better therapeutic outcomes.

eIF4E (Eukaryotic translation initiation factor 4E regulates protein translation in eukaryotes as a key factor. It is involved in the initiation and development of cancer by promote the aberrant expression of the proteins associated with tumors including Matrix metalloproteinases (MMPs) and VEGF [2]. VEGF-C (Vascular Endothelial Growth Factor C) belongs to the VEGF family as an important member. Acting on lymphatic endothelial cells (LECs), VEGF-C primarily interacts its receptor VEGFR-3 to promote cell growth and migration [3]. VEGF-C can also act directly on blood vessels to promote tumor angiogenesis and lymphangiogenesis, which might cause augmented metastasis [4]. E-cadherin is a member of cadherin (calcium-dependent adhesion) superfamily. The cadherins blong to a class of type-1 transmembrane proteinsand play important roles in cell adhesion via forming adherent junctions to bind cells within tissues together, thus, the E-cadherin - catenin complex plays a key role in cellular adhesion in tumor, loss of this function has been associated with tumour metastasis [5]. Matrix metalloproteinase-2 (MMP-2), called as 72 kDa type IV collagenase and gelatinase A, can promote the tumor invasion and metastasis by degrading type IV collagen wihcih is the most abundant component of the basement membrane [6].

The study aimed to investigate the expression, clinical significance and function of eIF4E, VEGF-C, E-cadherin, and MMP2 in colorectal cancer and determine the potential of eIF4E, VEGF-C, E-cadherin and MMP2 as indicators of colorectal cancer.

## RESUTLS

### The expression of eIF4E, VEGF-C, E-cadherin and MMP-2 in colorectal cancer

**Figure 1:**
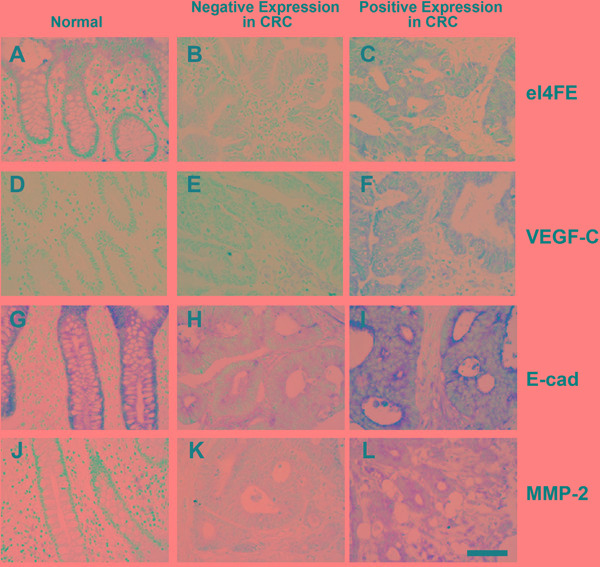
The expression of eIF4E, VEGF-C, E-cadherin and MMP-2 in colon cancerous tissues **A.** The expression of eIF4E in normal colon tissues around cancer. **B.** The negative expression of eIF4E in colon cancerous tissues. **C.** The positive expression of eIF4E in colon cancerous tissues. **D.** The expression of VEGF-C in normal colon tissues around cancer. **E.** The negative expression of VEGF-C in colon cancerous tissues. **F.** The positive expression of VEGF-C in colon cancerous tissues. **G.** The expression of E-cadherin (E-cad) in normal colon tissues around cancer. **H.** The negative expression of E-cad in colon cancerous tissues. **I.** The positive expression of E-cad in colon cancerous tissues. **J.** The expression of MMP-2 in normal colon tissues around cancer. K. The negative expression of MMP-2 in colon cancerous tissues. L. The positive expression of MMP-2 in colon cancerous tissues. Bar = 50 μM.

eIF4E proteins was found to mainly locate in cytoplasm of cancer (Figure 1A–1C). Through IHC followed by statistical analysis, the percentage of eIF4E positive cases in all patients with colorectal cancer was 91.7% (99/108, Table 1). However, only 10% (2/20) of adjacent normal tissues exhibited eIF4E signal, the eIF4E positive cases is only 10% in adjacent tissuses of cancer. The proteins of VEGF-C also mostly located in cytoplasm (Figure 1D–1F). Further histological and statistical analysis showed that 75.9% (82/108) patients with colorectal cancer were VEGF-C positive while only 4 cases were VEGF-C positive in adjacent tissuses. IHC images showed that E-cadherin mainly expressed in the membranes of intestinal mucosa epithelial cells of adjacent normal tissues (Figure 1G). In malignant tumor tissues, its locations were changed, expressing in both cytoplasm and partial membranes (Figure 1H and 1I). Further histological and statistical analysis showed that only 51.9% (56/108) of patients with colorectal cancer exhibited the expression of E-cadherin whereas 100% (20/20) of adjacent tissues were E-cadherin positive in membrane of epithelial cells. MMP-2 proteins mainly locate in the cytoplasm of cancer cells (Figure 1J-1L). Among 108 patients with colorectal cancer, 75.9% (82/108) of cases were MMP-2 positive. Meanwhile, only 35% of (7/20) cases were MMP-2 positive in adjacent tissues.

**Table 1:** Relationship between the expression of eIF4E, VEGF-C, E-cadherin and MMP-2 as well as clinical pathological parameters

Pathological parameters	n	eIF4E positive			VEGF-C positive			E-cadherin positive			MMP-2 positive		
		n	X^2^	P	n	X^2^	P	n	X^2^	P	n	X^2^	P
**Age (year)**													
< 50	28	24	1.299	0.254	21	0.017	0.894	16	0.434	0.515	23	0.799	0.371
≥50	80	75			61			40			59		
**Sex**													
Male	70	66	1.786	0.181	55	0.762	0.382	33	1.767	0.184	50	2.201	0.138
Female	38			33	27			23			32		
**Tumor Differentiation**													
Low	45	39	2.552	0.112	35	0.145	0.704	12	17.33	< 0.01	37	1.673	0.196
Moderate, High	63	60			47			44			45		
**The Depth of Invasion**													
In serosa	35	30	2.402	0.121	19	13.27	**<0.01**	23	3.986	**0.046**	22	4.838	**0.028**
Outside of serosa	73	69			63			33			60		
**Lymph Node Metastasis**													
Yes	82	80	15.49	**<0.01**	70	16.61	**<0.01**	35	10.183	**<0.01**	68	9.133	**<0.01**
No	26	19			12			31			14		
**Tumor Stage**													
A+B	26	19	15.49	**<0.01**	9	31.97	**<0.01**	29	9.857	**<0.01**	15	6.229	**0.013**
C+D	82	80			73			27			67		

### The relationship between the expression of eIF4E, VEGF-C, E-cadherin, MMP-2 and the clinical pathological parameters

The up-regulation expression frequency (EF) of eIF4E in colorectal cancer tissues was not associated with sex, age, tumor differentiation and the depth of tumor invasion (p>0.05); However, it is associated with clinical stage, and lymph node metastasis (p<0.05) (Table 1). The EF of VEGF-C in colorectal cancer tissues was not associated with sex, age of patients and tumor differentiation (p>0.05). However, it was associated with clinical stage, lymph node metastasis and the depth of tumor invasion (p<0.05) (Table 1). Interestingly, statistical analysis also showed that the expression frequency of E-cadherin in colorectal cancer was not associated with age and sex of patients (p>0.05) while it was associated with tumor differentiation, the depth of tumor invasion, lymph node metastasis and clinical stage (p<0.05). Similarly, the EF of MMP-2 in colorectal cancer was not associated with age and sex of patients and tumor differentiation (p>0.05) while it was strictly associated with the depth of tumor invasion, lymph node metastasis and clinical stage (p<0.05) (Table 1).

### eIF4E, VEGF-C, MMP-2 shortened and E-cadherin prolonged survival in patient-derived colon cancer xenografts

From human colon tumors, total 52 surgical specimens were isolated and s.c. implanted into NSG mice, a versatile immunodeficient strain from the Jackson Laboratory. After 4 weeks, 3 surgical specimens successfully caused tumor growth at the site of graft and were sustained by successive transplantation for up to the 10 passages. These patient tumor samples are determined as eIF4E, VEGF-C, MMP-2 and E-cadherin positive or negative, respectively. Their corresponding xenografts are referred as eIF4E positive and negative, VEGF-C positive and negative, MMP-2 positive and negative, and E-cadherin positive and negative, respectively. All xenografts went through an initial phase of tumor regression immediately after transplantation, followed by the reappearance of a palpable tumor and progressive tumor growth. The growth of engrafted tumors showed significant difference. This heterogeneity in growth rate probably reflects inherent patient-to-patient differences. We also examined the lung, heart, liver and kidneys of mice, when the tumor mass reached the maximum size deemed ethical before killing to search for metastases, but none were found (data not shown).

**Figure 2:**
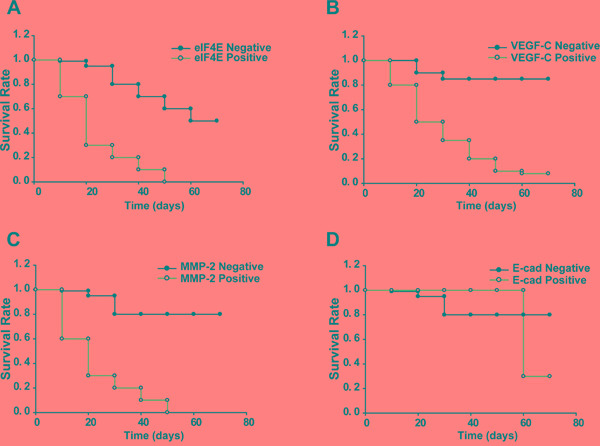
Kaplan-Meier survival analysis for xenograft mice Log rank test χ2= 75.0054. p< 0.0001. Fragments of human colon tumors were implanted subcutaneously (s.c.) into nude mice, and mice with tumors that reached 4–5 mm in diameter (n=5 per group) were applied to survival statistics. **A.** Kaplan–Meier survival curves of mice bearing eIF4E negative and positive tumors. **B.** Kaplan–Meier survival curves of mice bearing VEGF-C negative and positive tumors. **C.** Kaplan–Meier survival curves of mice bearing MMP-2 negative and positive tumors. **D.** Kaplan–Meier survival curves of mice bearing E-cadherin negative and positive tumors.

After establishing colon cancer xenografts from patients who had undergone surgery for colorectal cancer in nude mice, we evaluated the survival time of mice with different tumors. The mean survival times of mice with eIF4E positive and negative tumors were 26 and 45 days, respectively. The mice with eIF4E negative tumors prolonged the survival time compared with mice with eIF4E positive tumors (P<0.05; Figure 2A). Interestingly, the mean survival times for mice with VEGF-C positive and negative tumors were 30 and 68 days, respectively. The mice with VEGF-C negative tumors prolonged the survival time compared with mice with VEGF-C positive tumors (P<0.05; Figure 2B). Similarly, the mean survival times for mice with MMP-2 positive and negative tumors were 20 and 68 days, respectively. The mice with MMP-2 negative tumors prolonged the survival time compared with group with MMP-2 positive tumors (P<0.001; Figure 2C). However, the mean survival times for mice with E-cadherin positive and negative tumors were 60 and 65 days, respectively. The mice with E-cadherin positive tumors did not prolong the survival time compared with mice with E-cadherin negative tumors (P>0.05; Figure 2D).

### The expression of eIF4E, VEGF-C, MMP-2 and E-cadherin in primary site of patient-derived colon cancer xenografts

**Figure 3:**
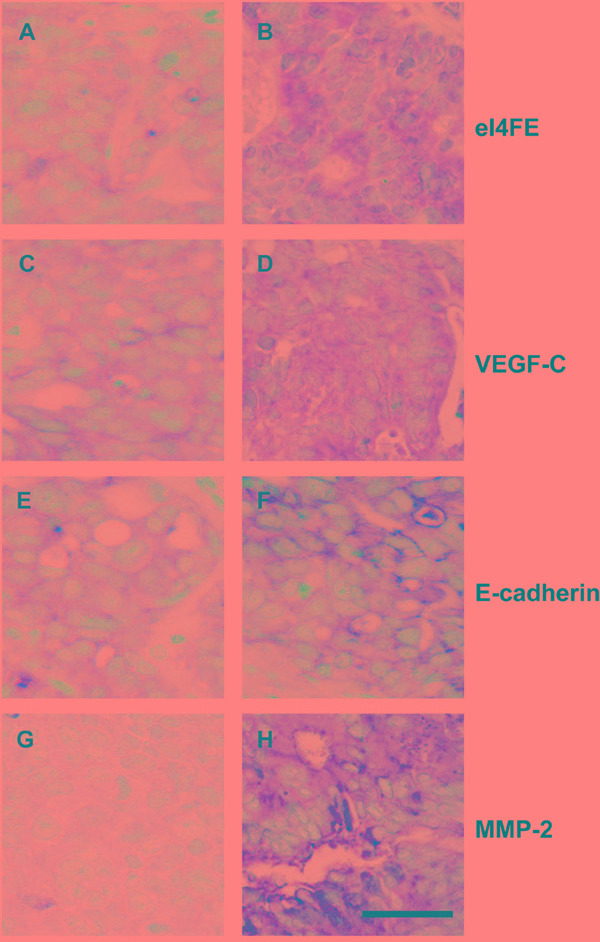
The expression of eIF4E, VEGF-C, MMP-2 and E-cadherin in primary site of patient-derived colon cancer xenografts **A.** The negative expression of eIF4E in colon cancerous tissues. **B.** The positive expression of eIF4E in colon cancerous tissues. **C.** The negative expression of VEGF-C in colon cancerous tissues. **D.** The positive expression of VEGF-C in colon cancerous tissues. **E.** The negative expression of E-cadherin in colon cancerous tissues. **F.** The positive expression of E-cadherin in colon cancerous tissues. **G.** The negative expression of MMP-2 in colon cancerous tissues. **H.** The positive expression of MMP-2 in colon cancerous tissues. Bar = 25 μM.

We furthermore investigated the expression of eIF4E, VEGF-C, MMP-2 and E-cadherin in primary site of patient-derived colon cancer xenografts. In each xenograft group, 2-3 mice were selected randomly and euthanized before natural death, tumors in primary implanted site were embedded in paraffin and IHC were performed to test the expression of eIF4E, VEGF-C, MMP-2 and E-cadherin. Compared with eIF4E negative xenograft group, the expression of eIF4E was still higher in eIF4E positive xenograft group. Similar results were validated in VEGF-C, MMP-2 and E-cadherin negative and positive xenograft groups (Figure 3).

### eIF4E, VEGF-C, MMP-2 promoted and E-cadherin suppressed the cancer cell growth

**Figure 4:**
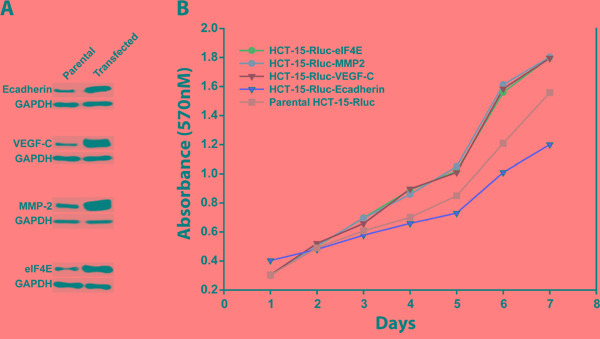
The growth rates of colon cancer cell lines HCT-15/Rluc with overexpression of E-cadherin, MMP-2, VEGF-c and eIF4E and parental HCT-15/Rluc **A.** Western blotting showed that the stable cell lines including HCT-15/Rluc/E-cadherin, HCT-15/Rluc/VEGF-C, HCT-15/Rluc/MMP-2, and HCT-15/Rluc/eIF4E were constructed successfully. **B.** The stable colon cancer cell lines with overexpression of E-cadherin, MMP-2, VEGF-c and eIF4E as well as parental HCT-15/Rluc cell line showed different growth rates.

Utilizing the stable HCT-15/Rluc cell lines (HCT-15/RLuc/eIF4E, HCT-15/Rluc/VEGF-C, HCT-15/Rluc/MMP-2, HCT-15/Rluc/E-cadherin and parental HCT-15/Rluc, Figure 4A), we investigated the effect of eIF4E, VEGF-C, MMP-2 and E-cadherin on the colon cancer cell growth. MMT assays showed that HCT-15/RLuc/eIF4E, HCT-15/Rluc/VEGF-C, HCT-15/Rluc/MMP-2 grew faster than parental HCT-15/Rluc colon cancer cell line (Figure 4B). The result suggested that overexpression of eIF4E, VEGF-C, and MMP-2 promote the colon cancer cell growth. Interestingly, HCT-15/Rluc-E-cadherin grew more slowly than parental HCT-15/Rluc cell line (Figure 4B), which suggested stable overexpression of E-cadherin inhibited the growth of colon cancer cells.

### eIF4E, VEGF-C, MMP-2 promoted and E-cadherin hampered the lung metastasis of colon cancer

**Figure 5:**
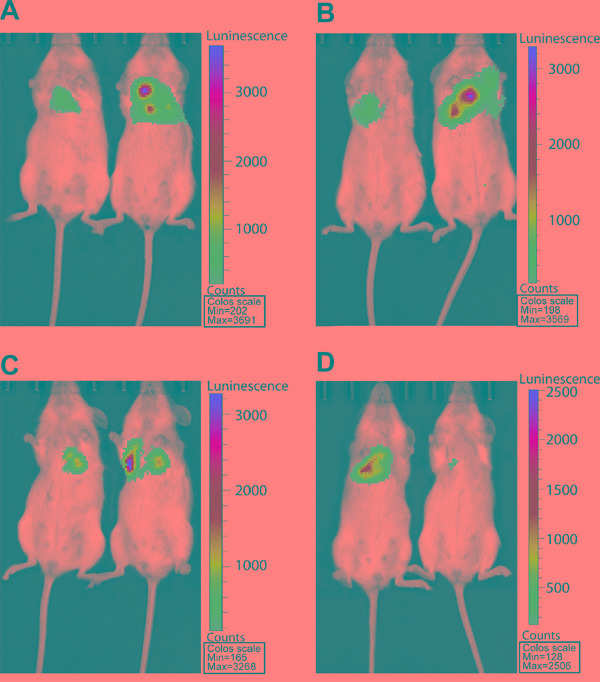
RL bioluminescence from *HCT-15/Rluc* cells present in Lung tissue in living mice **A.** The HCT-15/Rluc/eIF4E cells (1.0 × 10^6^) and corresponding HCT-15-Rluc were injected via tail-vein 3 days later. The bioluminescence seen represents the thorax region of the mouse where HCT-15/Rluc and HCT-15/Rluc/eIF4E cells are trapped in the lungs. **B.** The HCT-15/Rluc/VEGF-C cells (1.0 × 10^6^) and corresponding HCT-15/Rluc were injected via tail-vein 3 days later. The bioluminescence seen represents the thorax region of the mouse where HCT-15/Rluc and HCT-15/Rluc/VEGF-C cells are trapped in the lungs. **C.** The HCT-15/Rluc/MMP-2 cells (1.0 × 10^6^) and corresponding HCT-15/Rluc were injected via tail-vein 3 days later. The bioluminescence seen represents the thorax region of the mouse where HCT-15/Rluc and HCT-15/Rluc/MMP-2 cells are trapped in the lungs. **D.** The HCT/15-Rluc/E-cadherin cells (1.0 × 10^6^) and corresponding HCT-15-Rluc were injected via tail-vein 3 days later. The bioluminescence seen represents the thorax region of the mouse where HCT-15/Rluc and HCT-15/Rluc/E-cadherin cells are trapped in the lungs.

We next investigated the effect of eIF4E, VEGF-C, MMP-2 and E-cadherin on lung metastasis of colon cancer cells in mouse model, which mimics the clinical status of tumor metastases to distant sites. The mice were infused with HCT-15/RLuc/eIF4E, HCT-15/Rluc/VEGF-C, HCT-15/Rluc/MMP-2, HCT-15/Rluc/E-cadherin and parental HCT-15/Rluc stable cell lines through the tail vein injection. After 12 weeks of injection, bioluminescence (BLI) was performed to show the growth of lung metastatic tumors. BLI were stronger and BLI regions were larger in the thorax regions of the mice injected with HCT-15/RLuc/eIF4E, HCT-15/Rluc/VEGF-C, HCT-15/Rluc/MMP-2 than those in the mice injected with parental HCT-15/Rluc, (Figure 5A, 5B, 5C). The result suggested that eIF4E, VEGF-C, MMP-2 promote the lung metastasis of colon cancer. Excitedly, BLI was weaker and BLI region was smaller in the thorax regions of mice injected with HCT-15/Rluc/E-cadherin than those in the mice injected with parental HCT-15/Rluc stable cell line, which illustrated that E-cadherin limited the lung metastasis of colon cancer (Figure 5D).

### eIF4E regulated the expression of VEGF-C, MMP-2 and E-cadherin in colon cancer cells

**Figure 6:**
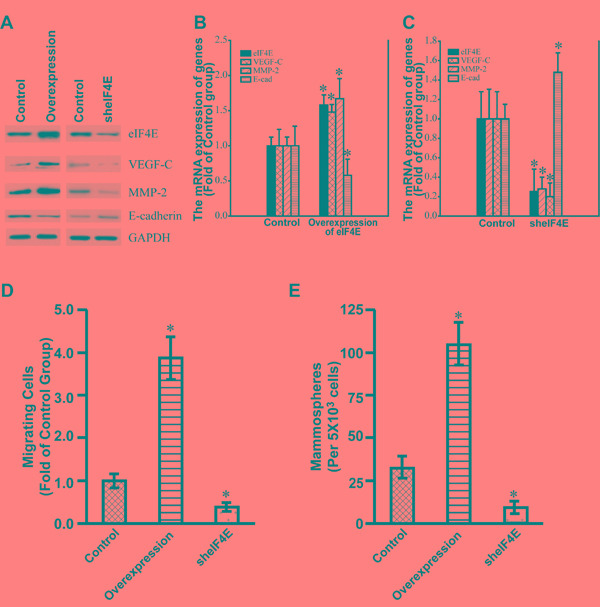
The expression of VEGF-C, MMP-2 and E-cadherin in the stable SW480 cell lines with the overexpression and Knockdown of eIF4E **A.** Western blotting showed the expression of eIF4E, VEGF-C, MMP-2, and E-cadherin in the corresponding stable SW 480 cell lines with the overexpression and knockdown of eIF4E. Control, Control stable SW480 cell lines by use of lentiviral infection packaged with control empty vectors. Overexpression, the stable SW480 cell line with the overexpression of eIF4E. sheIF4E, the stable SW480 cell lines with knockdown of eIF4E. The cells were lysed for loading on SDS-PAGE. The blotting were performed by use of indicated antibodies. **B.** Q-PCR showed the expression of eIF4E, VEGF-C, MMP-2, and E-cadherin in the stable SW 480 cell lines with the overexpression of eIF4E. **C.** Q-PCR showed the expression of eIF4E, VEGF-C, MMP-2, and E-cadherin in the stable SW 480 cell lines with the knockdown of eIF4E. Control, Control stable SW480 cell lines by use of lentiviral infection packaged with control empty vectors. Overexpression of eIF4E, the stable SW480 cell line with the overexpression of eIF4E. sheIF4E, the stable SW480 cell lines with knockdown of eIF4E. *, p<0.05. **D.** Relative number of migrating cells (*y*-axis) in a transwell migration assay is shown for Control, eIF4E-overexpression, and eIF4E-knockdown SW 480 cells. *, p<0.05. **E.** Number of mammospheres (*y*-axis) formed by Control, eIF4E-overexpression and eIF4E-knockdown SW480 cells. *, p<0.05.

To investigate the relationships between eIF4E and VEGF-C, MMP-2 as well as E-cadherin, we constructed stable colon cancer cell (CRC) lines by lentivrial infection of SW 480. Firstly, the stable SW 480 cell line with the overexpression of eIF4E was successfully constructed (Figure 6). The stable cell line showed the significant up-regulation of VEGF-C and MMP-2 compared with control cell line while the expression of E-cadherin was down-regulated significantly (Figure 6A, 6B). Secondly, we also constructed stable SW 480 cell line with the knockdown of eIF4E (Figure 6). The knockdown of eIF4E decreased the expression of VEGF-C and MMP-2, however, the knockdown of eIF4E increased the expression of E-cadherin (Figure 6A, 6C). These data demonstrated that eIF4E determined the expression of VEGF-C, MMP-2 and E-cadherin in CRC cell line SW480.

### eIF4E promoted the metastasis of colon cancer cells

To test whether eIF4E can promote the metastasis capacity and self-renewal of colon cancer cells, the Transwell migration assay and mammosphere formation assay were performed. We found that the overexpression of eIF4E significantly promoted the metastasis and self-renewal of the colon cancer cell line SW480 (Figure 6D, 6E). In addition, the knockdown of eIF4E significantly decreased the metastasis capacity and self-renewal ability of SW480 (Figure 6D, 6E). These data revealed that eIF4E stimulated the metastasis of colon cancer cells as an oncogenic factor.

## DISCUSSION

Metastasis is the principal cause for resulting in mortality of patients with colon cancer. Currently, most reports have demonstrated that cancer metastasis involves many complicated mechanisms including cell adhesions, cell connections, EMT (epithelial-mesenchymal transition), angiogenesis and organ-oriented metastasis [7]. Therefore, to investigate the regulating factors for tumor metastasis is the key to disclose the mechanism of the lethal process. As a key component of the eIF4F complex, eukaryotic translation initiation factor 4E (eIF4E is a crucial factor to initiate the protein translation. Normally, eIF4E expresses in a low level to maintain the activities of the translation of mRNAs. The aberrant increase of eIF4E activity results in the tumor initiation, development and metastasis through promoting the translation and expression of many oncogenes. Thus, eIF4E has been proposed to be an oncogene and has been proved to show augment in expression in malignant tumors of many organs such as breast, lung, cervix and colon [8–11]. Moreover, it is also closely related to lymph node metastasis and recurrence of tumor [12]. In the study, we investigated the expression of eIF4E in 108 colorectal carcinoma and adjacent normal tissues. The results showed that the percentage of the eIF4E positive cases was 91.7%. However, the eIF4E positive cases only occupied 10% in adjacent normal tissues. Further analysis showed that eIF4E was closely associated with the depth of invasion, lymph node metastasis and clinical stages. We also investigated the function of eIF4E in the metastasis of colon cancer *in vivo* and *in vitro*. The results showed that eIF4E was able to promote the metastasis of colon cancer cells both *in vivo* and *in vitro*. Our results herein demonstrated that eIF4E was a protein associated with colorectal carcinoma metastasis and played a key regulatory role in the lethal metastasis process of the tumor.

VEGF-C has been suggested to be the most important factor in promoting tumor metastasis via its function in lymphangiogenesis [4]. It has been demonstrated to be up-regulated in many cancers including colorectal adenocarcinoma [13–15]. In the report, we found that the percentage of VEGF-C positive cases was 75.9% in colorectal carcinoma while it was only 20% in adjacent tissues. Further analysis showed that the expression of VEGF-C was closely associated with the depth of invasion, lymph node metastasis and clinical stages. Furthermore, VEGF-C promoted the lung metastasis of colon cancer cells. The results presented herein demonstrated that VEGF-C is also a key regulator in the initiation and the lethal metastasis process of the colon cancer.

To forward invasion and metastasis, the tumor cells initially break through the limitation of cell adhesions. The degradation of basement membranes and stromal extracellular matrix (ECM) plays a critical role in the lethal process. The matrix metalloproteinases 2 (MMP-2, gelatinase A, 72 kDa) efficiently degrades components of the basement membrane and ECM such as Type IV collagen and fibronectin, and contributes to the invasion and metastasis of cancer cells [16–18]. In uterine leiomyosarcoma, MMP-2 has been found to be up-regulated and to be regarded as a key positive factor of tumor invasion and metastasis [19]. In the report, we also found that the up-regulation frequency of MMP-2 in cancerous tissues was higher (75.9%) than that in adjacent tissues (35%). The aberrant high expression of MMP-2 was closely associated with tumor differentiation, the depth of invasion, lymph node metastasis, and tumor stage. Furthermore, MMP-2 was confirmed to promote the lung metastasis of colon cancer cells. The results herein suggested that MMP-2 should be a protein associated with colorectal carcinoma (CRC) and promote CRC metastasis. Contrary to MMP-2, E-cadherin participates in cell adhesions through the E-cadherin-catenin complex. Down-regulation of E-cadherin expression is associated with tumor invasion and results in poor prognosis in many human malignancies including colon cancer [20–25]. Down-regulation of E-cadherin in colorectal adenocarcinoma have been demonstrated to be related to tumor growth and development [24]. In our report, E-cadherin was found to be expressed 51.9% in colorectal carcinoma. Furthermore, the staining of E-cadherin in cell membranes was weak and irregular. The result herein suggested that the down-regulation and aberrant expression of E-cadherin should be closely associated with the initiation and invasion of colon cancer. Further analysis showed that expression frequency of E-cadherin was closely associated with tumor differentiation, the depth of invasion, lymph node metastasis and tumor stages. The expression frequency of E-cadherin is higher in the colon cancer tissues with low differentiation, low invasion, low stages and no lymph node metastasis than those with high differentiation, deep invasion, high stage and lymph node metastasis. Lung metastasis of HCT-15/Rluc/E-cadherin colon cancer cell line showed that E-cadherin could suppress the lung metastasis of colon cancer cells. Thus we concluded that E-cadherin played a key suppressing role in the lethal metastasis process of the colorectal carcinoma.

The metastasis of tumors involve many proteins that facilitate angiogenesis (e.g. VEGF), invasion (e.g. MMPs), and cell survival and autocrine growth stimulation. In addition, the expression of these key malignancy-related proteins was regulated by eIF4E because eIF4E is a key regulator of the translation of these proteins [26]. It has been demonstrated that eIF4E positively regulated the expression and oncogenic roles of VEGF in a HNSCC cell line [27]. More interestingly, eIF4E also regulates the expression of MMP-2 in colorectal cancer. Down-regulation of eIF4E suppresses the expression of VEGF, MMP-2 and MMP-9 simultaneously in colon cancer [28]. Meanwhile, it has been reported that eIF4E negatively regulated the expression of E-cadherin in melanoma [29]. Figure 6 also showed that eIF4E regulated and determined the expression of VEGF-C, MMP-2 and E-cadherin in CRC cell lines. The results suggested that eIF4E may be taken as potential target for colon cancer treatment.

In conclusion, tumor metastasis involves the cooperative function of numerous proteins related to angiogenesis, invasion, loss of cell adhesion, cell survival. Here, we reported several proteins including eIF4E, VEGF-C, E-cadherin and MMP-2 which participate in the translation regulation of oncogenes, angiogenesis, and the loss of cell adhesions, respectively. We investigated their expression in 108 patients with colon cancer and analyzed their relationship in expression as well as the relationship between their up-regulation or down-regulation frequency and clinical parameters of patients. We further studied and validated their functions in metastasis both *in vivo* and *in vitro*. We found that eIF4E, VEGF-C and MMP-2 were up-regulated in colon cancer, and their expression frequencies are higher in cancerous tissues than in normal control tissues. During the metastasis, the expression frequency of E-cadherin is lower in cancerous tissues than in normal control tissues. Similarly, eIF4E, VEGF-C, and MMP-2 promoted and E-cadherin suppressed the lung metastasis of cancer cells. Among these protein factors, eIF4E dominates the regulation of VEGF-C, MMP-2 and E-cadherin, and is a key factor for colon cancer metastasis [30]. It is suggested that the regulation of eIF4E should be a potential target for preventing colon cancer invasion and metastasis.

## MATERIALS AND METHODS

### Patients and tissue specimens

Tissue specimens were obtained from 108 patients (70 males and 38 females) who underwent surgical resection due to colorectal carcinomas diagnosed at Chongqing Medical University between January, 2010 and December, 2012 (Table 1). The age of the patients ranged from 19-80 years old (mean, 59 years old). Based on the International Classification of Diseases for Oncology (ICD-O-3 Histology Coding), there were 63 cases of moderately-differentiated carcinomas and 45 cases of poorly-differentiated tumors (Table 1). Depth of invasion: 35 cases in serosa, 73 cases outside of serosa (Table 1). The clinical stage of colorectal adenocarcinoma including 9 cases of Stage A, 17 cases of Stage B, 53 cases of Stage C, and 29 cases of Stage D was classified based on China Dukes system. After surgery, 82 cases were with lymph node metastasis and 26 cases were without lymph node metastasis (Table 1). All patients involved in the study received no treatment prior to surgical resection. The resected tumors were examined histopathologically with the use of standard hematoxylin-eosin staining. 20 cases of Paraffin-embedded sections derived from cancer adjacent tissues diagnosed as normal colon tissues were involved in the study as normal control. These human studies have been performed in agreement with the ethical standards laid down in the 1964 Declaration of Helsinki and its latest revision in 2000 (the approval by the ethics committee of Chongqing Medical University, Chongqing, China). Informed consent was obtained from all patients.

### Immunohistochemistry

The paraffin-embedded tissue sections were subjected to immunostaining with the use of polyclonal antibodies against eIF4E, VEGF-C, and MMP-2 (Santa Cruz Biotechnology, Inc., Santa Cruz, CA, USA) and mouse monoclonal antibodies against E-cadherin (Santa Cruz Biotechnology, Inc.). The primary antibody was diluted in phosphate-buffered saline with 1.5% normal blocking serum. A streptavidin-biotin-peroxidase complex technique was used to reveal antibody-antigen reactions (LSAB kit; Dako, Glostrup, Denmark). Immunohistochemistry was performed following standard procedure. The slides were counterstained with hematoxylin. The evaluation of immunostaining for the studied protein was analyzed in 10 different tumor fields and the mean percentage of tumor cells with positive staining was scored. The cases were divided into positive and negative in terms of the analyzed markers. The presence of an immunohistochemical reaction in ≥10% of cells was considered a positive reaction, while a reaction in <10% was considered a negative reaction. Additionally, groups with a weak and strong reaction were formed from the cases of positive reactions (immunohistochemical reaction in <50% and >50% of cells, respectively).

### Establishment of tumor xenografts

The tumor xenografts were constructed following the protocol of He group [31]. Tumor tissues were sliced into 3~4 mm fragments. Then, the freshly obtained surgical specimens were implanted subcutaneously (s.c.) into 8-10 NSG mice (NOD.Cg-Prkdcscid Il2rgtm1Wjl/SzJ, denoted NSG in the report) within 2 h after the specimens were collected during surgery. After the tumors in these mice grew to 1 cm in diameter, they were collected and implanted into new mice to produce multiple generations. After the second passage, a mouse with a 1–2-cm tumor was used as a donor to implant tumors into other experimental mice. Mice were assigned randomly for tumor xenografts and were monitored for survival measurement. In the each group, 2-3 mice were randomly selected and euthanized, the tumors were embedded in paraffin, and IHC with described antibodies in the reported were performed. The mice involved in the experiments were housed under sterile conditions in a pathogen-free environment and provided with sterile water and food *ad libitum*. All manipulations were carried out aseptically inside a laminar flow hood. Animal experiments were carried out in compliance with the protocols approved by the Animal Welfare Committee of the Center for Laboratory Animal Medicine and Care at Chongqing Medical University.

### Noninvasive bioluminescence imaging of experimental lung metastases in vivo

Colon cancer cells and vector stably expressing luciferase (HCT-15/RLuc) were a gift from the University of Texas MD Anderson Cancer Center. Then we constructed colon cancer cells stably expressing luciferase as well as eIF4E, VEGF-C, MMP-2 and E-cadherin (HCT-15/RLuc/eIF4E, HCT-15/Rluc/VEGF-C, HCT-15/Rluc/MMP-2, and HCT-15/Rluc/E-cadherin). Cells were grown in RPMI 1640 supplemented with 10% FBS for 5 days. Then, cells were trypsinized, centrifuged, resuspended in 1x PBS, pH 7.4 and counted. 1×10^6^ cells in 200 μl of PBS were injected into the lateral tail vein of 10-week old female NOD/SCID mice (The Jackson Laboratory). Mice were monitored for lung metastases at 12 weeks after injection of cells using digital camera attached to computer with Living Image 4.4 software (IVIS Lumina XR; PerkinElmer, Waltham, MA). IVIS captures luminescence in the living animal. Mice were anesthetized with isoflurane/oxygen, and then luciferin (VivoGloTM Luciferin, In vivo Grade; Promega, Fitchburg, WI) was injected into the intraperitoneal cavity at 120 mg/kg (24 mg/ml). Images of the ventral side were collected for 1 min. Bioluminesce (BLI) in the lung region represents lung metastases. All animal in the experiments were performed in compliance with the protocols approved by the Animal Welfare Committee of the Center for Laboratory Animal Medicine and Care at Chongqing Medical University.

### Ectopic expression and knockdown of eIF4E in cell line

eIF4E cDNA was inserted into the eukaryotic retroviral expression vector pBabe. eIF4E and control siRNAs were purchased from Ribobio (Cat No Q000001977-1-B, Guangzhou, People's Republic of China). After screening, the siRNA sequence was inserted into retroviral expression vector pBabe too. SW480 cell line was infected by the lentivirus after packaging pBabe vectors as described previously [28] and screened by use of Paramycin to acquire stable cell lines. The overexpression and knockdown of eIF4E gene was tested by RT-qPCR 48 hours post-infection, and by Western blot 72 hours post-infection.

### Western blotting

Western blotting was conducted following the standard protocol. Protein lysates were prepared from cells in 1× lysis buffer (Cell Signaling Technology, Billerica, MA, USA), and protein concentration was measured with a proteinassay dye (Bio-Rad Laboratories, Hercules, CA, USA). Blots were probed with indicated primary antibodies, bound primary antibodies were detected with horseradish peroxidase conjugated secondary antibodies, and signals were visualized with enhanced chemiluminescent reagents (Thermo Scientific).

### MTT assay

Cells were plated at concentrations of 2.5 × 10^4^cells per well in 96-well plates and incubated for 7 days with either 100 μl of growth medium. Cell growth was measured using a 3-(4,5-dimethylthiazol- 2-yl)-2,5-diphenyl-tetrazolium bromide (MTT) assay (Sigma, MO, USA) by the addition of 20 μl of MTT solution (5 mg/ml, dissolved in sterile water) to each well and incubation for 4 h before the addition of 100 μl of solubilization buffer (10 % sodium dodecyl sulfate, 0.01 M HCl). Absorbance at 570 nm was measured the next day using Synergy 2 microplate reader with Gen5 software [32].

### Transwell migration assay

Transwell migration assay in CRC was performed following the protocol of Chang Lab with slight modification [33]. 2.5×10^5^ SW480 cells were seeded in 500 μM starvation medium (0.1% FBS) onto the upper chamber of a Thinsert Cell Culture Insert (Greiner Bio-One #665638) in a 12-well plate. The lower chamber was filled with complete growth media. Cells were allowed to migrate for 24 hr at 37°C and 5% CO2. Then, the unmigrated cells were removed from the top surface of the upper chamber by scrubbing it 2x with a wet cotton swab. The migrated cells were fixed on the bottom surface of the upper chamber with 100% methanol for 10 min at room temperature and washed twice with water. After staining with with DAPI (5 ng/ml) for 2 min, and washing twice with water. The migrated cells were counted by using a digital inverted microscope EVOS fl with fluorescence light application at 10x objective in 10 visual fields. The statistical analysis derived from independent triplicates.

### Mammosphere formation assay

We separated cell suspension into single cells using a 25 g needle. Then, the SW480 cells with control vector, eIF4E-overexpression Vector and sheIF4E Vector were seeded at a density of 5000 cells/well on ultra low attachment 6-well plates (Corning #07-200-601). Cells were cultured in 2 ml of mammosphere formation media, which is serum-free DMEM/F12 supplemented with 100 IU/ml penicillin, 100 μg/ml streptomycin, 10 ng/ml basic FGF, 20 ng/ml EGF, 2% B27 (Invitrogen). Cells with a three dimensional spherical structure (spheres) were collected 7 to 10 days later by gentle centrifugation (300g/min, 3min). All data are collected from three independent experiments.

### Statistical analysis

The statistical analysis was performed by use of GraphPad Prism 5 (GraphPad Software, USA). The differences of parameters between indexes were analyzed through χ^2^ test.. For survival data, Kaplan–Meier curves were plotted and compared using a log-rank test. All tests were two-sided. P values were adjusted for multiple comparisons using the Bonferroni method. *P<0.05* was used to indicate a statistically significant difference.
